# Zein Beta-Cyclodextrin Micropowders for Iron Bisglycinate Delivery

**DOI:** 10.3390/pharmaceutics12010060

**Published:** 2020-01-11

**Authors:** Diletta Esposito, Giovanni Dal Poggetto, Aurélie Demont, Nicolai Kraut, Agnese Miro, Francesca Ungaro, Paola Laurienzo, Fabiana Quaglia

**Affiliations:** 1 Drug Delivery Laboratory, Department of Pharmacy, University of Napoli Federico II, Via Domenico Montesano 49, 80131 Napoli, Italy; diletta.esposito@unina.it (D.E.); miro@unina.it (A.M.); ungaro@unina.it (F.U.); 2 Institute for Polymers, Composites and Biomaterials CNR, Via Campi Flegrei 34, 80078 Pozzuoli, Italy; giovanni.dalpoggetto@ipcb.cnr.it (G.D.P.); paola.laurienzo@ipcb.cnr.it (P.L.); 3 BÜCHI Labortechnik AG, Meierseggstrasse 40, 9230 Flawil, Switzerland; demont.a@buchi.com (A.D.); kraut.n@buchi.com (N.K.)

**Keywords:** zein, beta-cyclodextrin, powders, iron bisglycinate, food supplements

## Abstract

Given the limited number of materials available to design delivery platforms for nutrients, the rational combination of raw materials already approved as food ingredients and their processing through nano-micro technology can offer a unique tool for innovation. Here, we propose a nano-in-micro strategy to produce powders based on the hydrophobic protein zein, useful for the oral delivery of a hydrophilic iron source (iron bisglycinate) in anaemic patients. Iron-loaded powders were prepared through a two-step strategy consisting in the formation of a zein pseudolatex followed by a spray-drying step. To extend the manipulation space for zein and entrap iron bisglycinate, β-cyclodextrin (βCD) was selected as helping excipient. Addition of βCD allowed iron loading in the pseudolatex and greatly increased product yields after the drying process as compared to zein alone. Iron-loaded micro-sized powders were characterised by attenuated total reflectance–Fourier transform infrared (ATR-FTIR) spectra, thermogravimetric analysis (TGA), and differential scanning calorimetry (DSC) to elucidate the role of βCD as a compatibilizer for the zein–iron system. Remarkably, micropowders released only 20% of FeBIS in a simulated gastric fluid, whereas release in a simulated intestinal fluid was almost completed in 7 h. In summary, βCD association to zein is a novel strategy to expand applications in the oral delivery of iron bisglycinate and, prospectively, to micronutrient chelates.

## 1. Introduction

A significant trend in the modern food industry is the development of food supplements specifically designed to improve human health and well-being, reduce the incidence of certain diseases, and improve human performance. In this context, the design, fabrication, and characterisation of polymer-based delivery platforms able to protect and to deliver the bioactive components in the body are in the limelight at the moment [1,2].

Among natural polymers, zein is a corn protein that has been widely employed at an industrial level due to several advantages. As plant-based protein extracted from corn, it is a renewable source with broad acceptance compared to animal proteins [3], and it is one of the few hydrophobic water-insoluble biopolymers approved for oral use by Food and Drug Administration [4]. The renewed interest in the biomedical and pharmaceutical field is due to its peculiar properties and possibility to be used in different modified-release platforms such as films, microparticles, nanoparticles, gels, or as coatings for tablets/pellets [5]. The hydrophobicity of zein, attributed to the presence of more than 50% non-polar amino acids (leucine, alanine, and proline), results in poor solubility in water.

Corn zein (α type) is soluble in 50–90% water/ethanol depending on the composition of the raw material [6] and easily nano-precipitates from hydroalcoholic solution after the addition of water as anti-solvent. Thus, appropriate manipulation of experimental conditions allows the formation of zein nanoparticles loaded with hydrophobic bioactive components previously co-solubilized in the zein hydroalcoholic solution such as fish oil [7], α-tocopherol [8], vitamin D3 [9], daidzin [10], and curcumin [11,12]. On the other hand, the entrapment of hydrophilic compounds in zein-based nanoparticles is much more difficult to attain since their precipitation in the hydroalcoholic solution can occur, especially when high ratios between the bioactive molecule to load and the polymer are set. To this purpose, nanoparticle formation becomes much more complex, requiring multiple steps, which unavoidably makes the industrial scale-up challenging to attain. Another issue associated with zein nanoparticles is their poor stability in aqueous systems with pH close to the isoelectric point (pI) of zein (ca. 6.2) [12]. The zein nanoparticle (NP) surface-stabilized with proteins [13], polysaccharides [14], and other anionic polymers like gum arabic [15], pectin [16], and alginate [17] can be proposed to overcome stability issues because of the possibility of obtaining a dry product.

Combination of zein with different polymers may represent a valid strategy to enlarge the manipulation space for zein-based delivery platforms and to develop novel oral delivery systems [18]. In this regard, cyclodextrins are ideal candidates since they can form supramolecular complexes with hydrophobic amino acids and change protein properties [19]. In fact, cyclodextrins can inhibit protein aggregation, increase the thermal stability of liquid protein formulations, and act as stabilisers during the spray- and freeze-drying process [19]. Only β-cyclodextrin (βCD) is approved in Europe as an additive in food supplements, while different natural and semisynthetic cyclodextrins are already employed in marketed drugs [20].

The combined use of zein and cyclodextrins as delivery platforms for poorly water-soluble compounds has been scarcely explored so far. Zein/βCD antimicrobial fibres for food packaging [21,22,23], microparticles entrapping α-tocopherol able to ensure colour stability and shelf-life of fruit beverages [24], and powders delivering quercetin for oral bioavailability improvement [4] have been developed so far. To our knowledge, the development of strategies for the delivery of hydrophilic molecules has been minimal.

Iron is a trace mineral that is naturally present in many foods, is added to some foods to achieve fortification, and is available as a dietary supplement. Frequently used forms of iron in supplements include ferrous and ferric iron salts such as ferrous sulfate, ferrous gluconate, ferric citrate, and ferric sulfate. Because of its higher solubility, ferrous iron in dietary supplements is more greatly bioavailable than ferric iron [25]. Despite its wide diffusion, most oral iron supplements have been associated with erosive mucosal injury in the upper gastrointestinal tract as well as nausea, vomiting and epigastric discomfort, diarrhoea, and constipation [26]. Side effects, bad taste, low bioavailability, and possible interactions with other components in the mouth or multimineral/multivitamin supplements have all prompted the investigation of appropriate delivery strategies (chelation, encapsulation) [27]. 

Herein, we explore the applicability of zein as a base material for the delivery of iron. Iron-loaded micropowders were prepared through a two-steps nano-in-micro strategy consisting in forming a zein/βCD pseudolatex via antisolvent precipitation followed by a spray-drying step. Experimental conditions to obtain zein/βCD pseudolatexes and powders were preliminarily set and interactions between components clarified. After that, iron was incorporated at different loadings and the final powders fully characterised in the solid state and in simulated oral fluids to evaluate their potential in oral delivery.

## 2. Materials and Methods

### 2.1. Materials

Corn zein (Zein F4400C non-GMO/food grade) was a kind gift of Flo Chemical Corporation (Ashburnham, MA, USA). KLEPTOSE^®^ (beta-cyclodextrin, βCD) was purchased by Roquette Italia SpA (Alessandria, Italy) and iron bisglycinate (FeBIS, total iron content of 26% by wt) was a kind gift from Giusto Faravelli SpA (Milan, Italy). Phenolphthalein, sodium chloride, sodium hydroxide, sodium carbonate, monobasic potassium phosphate, and pepsin from porcine gastric mucosa were from Sigma-Aldrich (Milan, Italy). Methanol and hydrochloric acid were from Carlo Erba Reagents (Milan, Italy), and ethanol was from Honeywell (Seelze, Germany). All the other chemicals were of analytical reagent grade. Ultrapure water was used for all experiments. 

### 2.2. Production of Pseudolatexes

Zein pseudolatexes (PLs) were formed by the anti-solvent co-precipitation method [28], adding 50 mL of a water solution of βCD (0.5–1.5% *w*/*v*) to 50 mL of a solution of zein in ethanol/water 80% *v*/*v* at room temperature and under magnetic stirring. The following process parameters were tested: (1) zein and βCD concentration in the hydroalcoholic and water solution, respectively, and (2) the ratio between hydroalcoholic and water phases.

In the first set of experiments, βCD concentration was fixed at 1% *w*/*v* while zein was employed in a concentration range of 2–6% *w*/*v* while maintaining a 1:1 volume ratio between the two solutions.

In the second set of experiments, zein concentration was fixed at 4% *w*/*v* while βCD was used in a concentration range of 0.5–1.5% *w*/*v*. Pseudolatex of zein alone were prepared analogously using water instead of the βCD solution.

### 2.3. Characterisation of Pseudolatexes

The hydrodynamic diameter (D_H_) and polydispersity index (PI) of pseudolatex were assessed on a Zetasizer Nano ZS (Malvern Instruments Ltd., Malvern, UK). The amount of βCD embedded in the pseudolatex was evaluated indirectly from the amount of βCD remaining in the water solution after pseudolatex formation. βCD quantification was carried out following two protocols: (1) a spectrophotometric assay based on the shift of colour of a phenolphthalein solution upon βCD complexation, and (2) by weighting the lyophilised βCD-containing aqueous phase after pseudolatex centrifugation. In the first method, βCD was quantified by UV analysis of the fading of a phenolphthalein alkaline solution [29]. Phenolphthalein/βCD form a colourless stable inclusion complex (molar ratio 1:1) which was directly related to the amount of βCD added to the solution [30]. Briefly, a stock phenolphthalein solution in methanol (3 mM) was diluted 1:100 in 0.05 M carbonate buffer at pH 10.5 just before use. Then, 1.3 mL of the phenolphthalein working solution were added to 200 μL of the sample, and the absorbance of the resulting solution was immediately measured at 553 nm (phenolphthalein λmax) on an UV-1800 (Shimadzu Corporation, Tokyo, Japan). The linearity of the response was verified over the βCD concentration range of 0.1–1.00 mg/mL (*R*^2^ > 0.99). All the measurements were performed in triplicate at room temperature.

In the second method, the amount of unbound βCD was evaluated by weighting the mass of lyophilised supernatant obtained after the centrifugation of pseudolatex at 5400× *g* for 30 min.

Both methods were performed considering the interference of zein in the pseudolatex prepared without CD.

### 2.4. Production of Micropowders 

Micrometric powders were obtained by spray-drying the pseudolatex in a Büchi Mini Spray Dryer B-290 (BÜCHI Labortechnik AG, Flawil, Switzerland). Different process parameters were preliminarily tested on pseudolatex made only with zein to optimise the properties of the final products (yield, adhesiveness, flow properties). For pseudolatex including βCD the following operating conditions were maintained: (1) inlet drying temperature 115 °C, (2) outlet drying temperature 60 °C, (3) pump 10%, and (4) aspirator level 90%. After the drying process, the powders were collected, sieved, and stored at room temperature. The yield of the production process was calculated from the weight of collected micropowders.

FeBIS-loaded micropowders were produced by spray-drying a pseudolatex prepared from a zein hydroalchoholic solution at 4% *w*/*v* and a water solution containing βCD and FeBIS at 1% and 0.1% *w*/*v*, respectively. The volume ratio between phases was 1:1. The theoretical loading of the final powder was 2% *w*/*w* corresponding to 0.5% *w*/*w* Fe^2+^. It was not possible to form pseudolatex of FeBIS in the absence of βCD.

To maximise the theoretical loading of FeBIS, component concentrations and volume of the solutions needed to be changed. Zein was used at 2% *w*/*v* in the hydroalcoholic solution, βCD was set at 0.1% *w*/*v* and FeBIS at 0.04% *w*/*v*, respectively. The volume ratio between the hydroalcoholic and water phases was 1:5. The theoretical loading of the final powder was 8% *w*/*w*, corresponding to 2% *w*/*w* Fe^2+^.

### 2.5. Solid State Characterisation of Micropowders

Scanning electron microscopy (SEM) and energy dispersive X-ray (EDX) mapping analysis were performed to evaluate the morphology of micropowders and the distribution of iron in the solid, respectively. For SEM analysis, Quanta 200 FEG apparatus (FEI, Hillsboro, OR, USA) equipped with an Inca Energy 250 and an Inca-X-act LN2-free analytical silicon drift detector (Oxford Instruments, High Wycombe, UK) were used. Samples were coated with Au/Pd alloy. Micrographs were taken by using a beam intensity of 30 kV. For EDX analysis, a Phenom XL (Alfatest, Milan, Italy) was used. The distribution of iron was analysed with Phenom XL Desktop software.

The bulk/tapped density of the sieved powders was evaluated before/after a compaction process according to method 1 of the European Pharmacopoeia (9 ed. monograph 2.9.34., Bulk density and tapped density of powders). Accordingly, the flow properties of micropowders were estimated as per Carr’s Index. 

The evaluation of the amount of uncomplexed βCD in the micropowders was carried out using the same protocols described above for the pseudolatex. An amount of micropowder (100 mg) was treated with water (4 mL) to solubilise free βCD. After centrifugation (5400× *g* for 30 min), the supernatant was collected and either treated with phenolphthalein (1) or freeze-dried and weighted (2).

Fourier transform infrared (FTIR) analysis was performed with a Paragon 500 spectrometer (Perkin Elmer, Shelton, CT, USA) equipped with a ZnSe attenuated total reflectance (ATR) crystal accessory. Samples were placed in direct contact with the ATR crystal and pressed with a pressure clamp positioned over the crystal/sample area to allow intimate interaction between the material and the crystal. Spectra were acquired in the 4000–400 cm^−1^ range, at a resolution of 2 cm^−1^ (average of 20 scans).

NIR spectra were collected in reflectance mode with an NIRFlex^®^ N-500 FT-NIR spectrometer (BÜCHI Labortechnik AG, Flawil, Switzerland) over the range of 4000–10,000 cm^−1^, with 4 cm^−1^ resolution. The micropowders were analysed in vials with 8 mm outer diameter. The results were reported as the percentage of reflactance. 

Thermogravimetric analysis (TGA) was carried out on a Pyris Diamond TG-DTA (Perkin-Elmer, Shelton, CT, USA) apparatus from 25 to 400 °C under nitrogen flow (50 mL/min) at 10 °C/min heating rates. 

Differential scanning calorimetry (DSC) analysis was carried out on a Q2000 (TA Instruments, Lukens Drive, New Castle, DE, USA) under nitrogen flow. Samples (about 4 mg) sealed in an aluminium crucible were heated from 25 to 120 °C at 10 °C/min scanning rate, cooled to 25 °C at 20 °C/min and finally heated again up to 200 °C at 10 °C/min (second heating run). 

### 2.6. Zein Quantification in the Micropowders

This method was carried out to assess the amount of zein in raw material and spray-dried products. Zein raw material or micropowders (40–250 mg) were weighted using Kjeldahl weighting boats (nitrogen-free) and placed in Kjeldahl tubes (300 mL) adding three tablets of titanium-micro as the catalyst and 10 mL of 98% *w*/*v* H_2_SO_4_. All samples were digested in KjelDigester K-449 for 2 h at 420 °C (one digestion cycle). After digestion (samples were clear-green digestate), the samples were cooled to room temperature. Then the acidic digestion mixtures were diluted with distilled water (25 mL), and the samples tubes were transferred in the KjelMasterK-375 distillation unit (BÜCHI Labortechnik AG, Flawil, Switzerland). The digestion mixtures were alkalinised with NaOH 32% *w*/*v* (45 mL) before distillation to free-up ammonia. Ammonia was steam-distilled into an acidic receiver solution of H_3_BO_3_ 4% *w*/*v* with Sher indicator (60 mL). The nitrogen content was determined by the titration of the borate complex with H_2_SO_4_ 0.1 M according to Equation (1):

(1) $$\%\;N=\left[V\left(l\right)-V\left(\mathrm{Bl}\right)\right]\times F\times c\times f\times \frac{M\left(N\right)}{m\times 1000}\times 100$$

where % *N* is the % of weight of *N*, *V*(l) is the consumption of titrant, sample (mL), *V*(Bl) is the average consumption of titrant, blank (mL), *F* is the molar reaction factor (1 = HCl, 2 = H_2_SO_4_), *c* is the concentration of titrant (0.1 mol/L), *f* is the factor of titrant (1), *M*(N) is the molecular weight of N (14,007 (g/mol)), and *m* is the sample weight (g).

A protein factor (PF) of 6.25 was used to calculate the protein content (% P).

### 2.7. Release of Iron Bisglycinate from Micropowders

The release properties of the micropowders loaded with FeBIS was assessed in simulated gastric fluid (SGF) pH 1.2 (2 g NaCl, 80 mL of 1 M HCl, 3.2 g pepsin powder from porcine gastric mucosa in 1 L of water) and in simulated intestinal fluid (SIF) pH 6.8 (77 mL of 0.2 M NaOH, 6.8 g KH_2_PO_4_, 10 g of pancreas powder in 1 L of water) according to European Pharmacopeia indications. Fifty milligrams of micropowders were placed in 4 mL of SGF or SIF under stirring. Samples were taken at different time points and centrifuged at 16,300× *g* for 15 min. Iron content in the sample was assessed by inductively coupled plasma–mass spectrometry (ICP-MS). To this purpose, 100 μL of the supernatant were treated with 9.2 mL of ultrapure water and 0.7 mL of HNO_3_ 65% *w*/*v* and analysed with an Agilent 7500ce apparatus (Agilent Technologies, Inc., Santa Clara, CA, USA) with a collision/octopole reaction system (ORS) to reduce polyatomic interferences. Instrument performances were checked using proper Tuning Solution (AGILENT^®^) until the setting related to sensitivity and interference parameters were optimised. Possible interferences were tested through Interference Check Solutions (AGILENT^®^).

### 2.8. Statistics

Data are expressed as the mean ± standard deviation (SD) of at least three experiments. Post hoc paired Student’s *t*-test was used to investigate significant differences. In all cases, *p* < 0.05 (one-tail) was considered to be statistically significant. All data processing was performed using Excel statistics.

## 3. Results

### 3.1. The Issue of Iron Entrapment in Zein-Based Nanoparticles

As an iron source, we selected iron (II) bisglycinate (FeBIS), which is commonly employed in several food supplements. The general strategy proposed here to form zein powders consists in (1) preparing a stable colloidal dispersion (referred to as pseudolatex) through an anti-solvent precipitation method (first step), and (2) drying the pseudolatex by spray-drying to achieve a solid (second step). Experimental conditions were set to obtain pseudolatexes withstanding (1) size compatible with nebulisation process and (2) colloidal stability during spray-drying.

The most logical way to entrap FeBIS in the pseudolatex was to dissolve the iron salt in the hydroalcoholic zein solution and achieve particle hardening by adding water. Unfortunately, massive aggregation occurred at different zein/FeBIS concentrations (data not shown) due to the low solubility of FeBIS in the hydroalcholic solution. 

**Figure 1:**
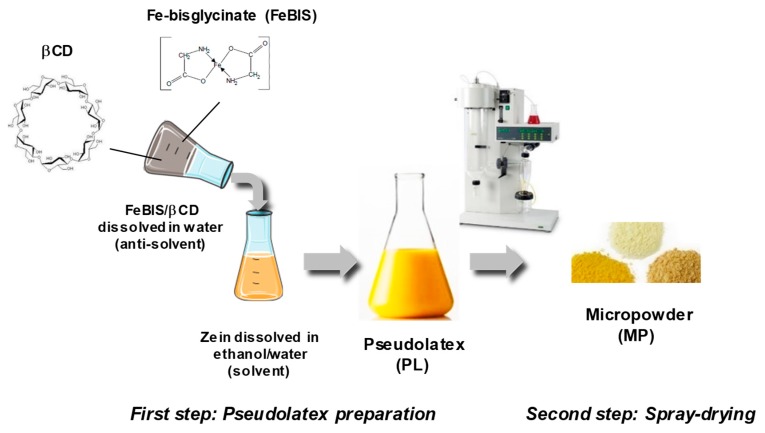
Schematic representation of the powder production process through the nano-in-micro strategy.

As an alternative, we decided to dissolve FeBIS in the antisolvent and achieve encapsulation upon mixing with the hydroalcoholic zein solution. Again, massive aggregation occurred at different zein concentrations and solvent/antisolvent ratios. Thus, we introduced βCD as helping excipient in the antisolvent to alter zein conformation/solubility and, in so doing, to make zein and FeBIS compatible from a chemical-physical standpoint. The general procedure to prepare FeBIS-loaded powders is illustrated in Figure 1.

A direct method to establish if a protein changes its conformation in the presence of an interacting species is to analyse UV and fluorescence spectra at different protein/complexant ratios. We found that βCD could interact with zein as demonstrated by the depression of UV maximum absorption for zein at 278 nm and a progressive decrease of baseline scattering (supplementary information, Figure S1a). A depression of the emission band with a maximum at 305 nm typical of tyrosine and tryptophan was observed in parallel (supplementary information, Figure S1b). 

### 3.2. First Step: Development of Zein/βCD Pseudolatex 

**Figure 2:**
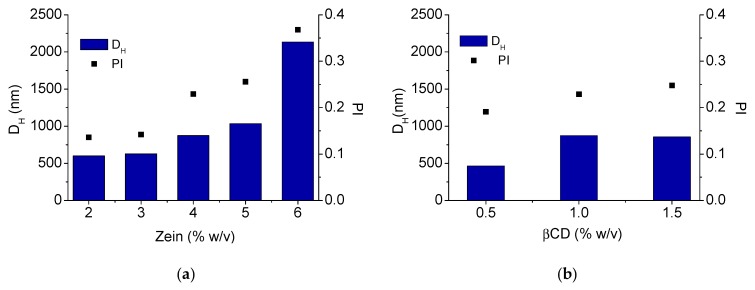
Size and polydispersity (PI) of zein/βCD pseudolatexes. Effect of zein concentration (2–6% *w*/*v*) at a fixed βCD concentration (1% *w*/*v*) (**a**); effect of increasing βCD concentration (0.5–1.5% *w*/*v*) at a fixed zein concentration (4% *w*/*v*) (**b**). D_H_: hydrodynamic diameter.

In the first part of the study, we investigated the impact of zein concentration and βCD addition on pseudolatex hydrodynamic diameter (D_H_) and polydispersity (PI) (Figure 2). In the formulation approach, the concentration of βCD in water was maintained constant at 1% *w*/*v* while zein concentration was gradually increased. As illustrated in Figure 2a, D_H_ and PI increased proportionally as the concentration of zein in the hydroalcoholic solution did. No macroscopic aggregation was also observed at large particle size and high PI. At each zein concentration tested, the addition of βCD 1% *w*/*v* in the aqueous phase did not impact significantly on the final size and PI (data not shown).

After that, the effect of increasing βCD amount at a fixed zein concentration (4% *w*/*v*) was evaluated (Figure 2b). Zein concentration of 4% *w*/*v* gave, in fact, a pseudolatex with satisfactory colloidal stability along time (no change of size observed up to 4 h). An increase of βCD concentration in the antisolvent aqueous phase up to 1.5% *w*/*v* (which is very close to maximum βCD solubility in water) resulted in a pseusolatex with D_H_ increasing from ca 500 nm to ca 800 nm, whereas PI remained in the range 0.20–0.25.

### 3.3. Second Step: Processing Pseudolatex by Spray-Drying 

This step focused on setting-up suitable operation conditions for spray-drying zein and corresponding zein/βCD pseudolatexes developed in 3.2 (compositions in Table S1). 

In a pilot experiment, a powder of zein (MP_Z_2_) was produced by employing different process parameters to optimise process conditions. As shown in Table S2, the maximum yield around 60% was achieved by increasing the inlet temperature, which did not increase further at a doubled pumping rate of the feeding liquid. In the absence of βCD, the yield of the process was never higher than 60%. Based on these preliminary results the inlet drying temperature was set at 115 °C which gave an outlet value in the range 55–60 °C (i.e., the maximum temperature encountered by the powder during the drying process). In these operating conditions, the drying process gave a solid with yields above 70%, reaching even 90% for MP_Z_2_/CD_0.5_. 

**Figure 3:**
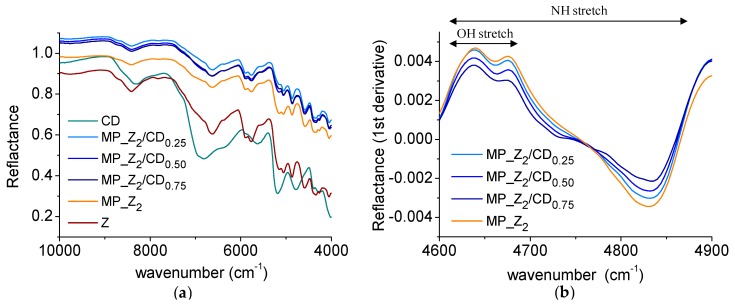
NIR spectra of zein and zein/CD micropowders. Reflectance (**a**) and first derivative spectra in the interval 4600–4900 cm^−1^ (**b**).

The produced powders were investigated by NIR to get preliminary insight in zein/βCD interactions. As evidenced in Figure 3a, a different shift in the spectral bands was found as a function of βCD amount in the sample. In particular, the OH/NH stretch region (Figure 3b), was changed depending on the βCD amount in the powder. 

The quantification of free βCD for MP_Z_2_/CD_0.5_ powder with the highest yield gave values around 50% of the total βCD employed (49 ± 5 and 49 ± 1 for βCD quantification via protocols 1 and 2 described in the experimental part). A similar amount of free CD was found in the aqueous phase of pseudolatex feeding (51 ± 11 and 45 ± 7 for βCD quantification via protocols 1 and 2 described in the experimental part) indicating that βCD is partly associated to zein matrix during pseudolatex formation and partly uncomplexed. 

The flow properties were estimated after the evaluation of the bulk/tapped density of the sieved powders. The presence of βCD did not affect significantly the flowability of the powder expressed as Carr’s Index (MP_Z_2_ = 38 ± 2.8%; MP_Z_2_/CD_0.25_ = 37 ± 4.8%; MP_Z_2_/CD_0.5_ = 37 ± 8.0%; MP_Z_2_/CD_0.75_ = 38 ± 2.8%).

### 3.4. Development of Zein/βCD/FeBIS Micropowders

**Figure 4:**
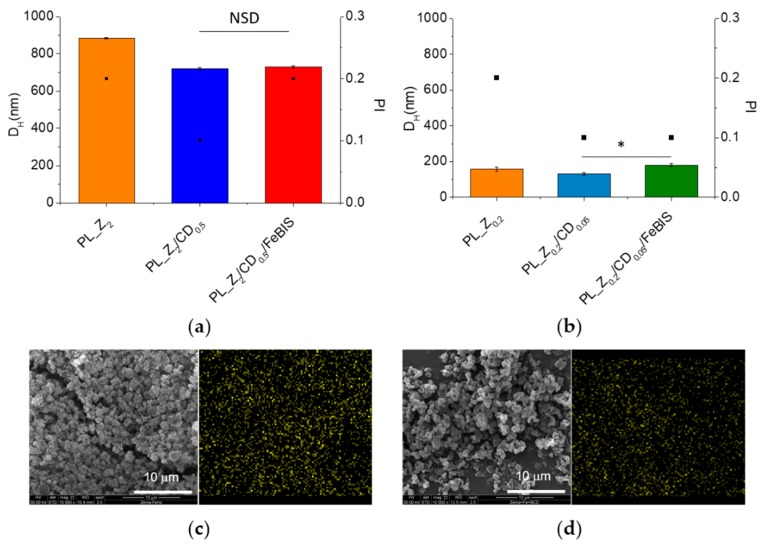
D_H_ (bars) and PI (squares) of the pseudolatex employed to prepare powders at FeBIS theoretical loading of 2% (**a**) and 8% *w*/*w* (**b**). SEM images and corresponding energy dispersive X-ray (EDX) mapping of iron (yellow) in the MP_Z_2_/CD_0.5_/FeBIS (**c**) and MP_Z_0_._2_/CD_0.05_/FeBIS (**d**). NSD: no significant difference. * *p* < 0.02.

FeBIS was first dissolved in the βCD water solution (Figure 1) and then added to the hydroalcoholic zein solution. The concentration of zein and βCD selected to prepare pseudolatex were those achieving a dispersion having a size compatible with nebulisation (<1 μm to avoid nozzle blocking) and at a high solid content for a shorter spray-drying process (PL_Z_2_/CD_0.5_). The maximum amount of FeBIS that could be loaded without causing massive aggregation of the pseudolatex was 2% *w*/*w* of the total solid. The D_H_ values of the FeBIS-containing pseudolatex at low FeBIS loading were unchanged as compared with those of unloaded pseudolatex (Figure 4a).

To increase FeBIS theoretical loading, we needed to optimise formulation again. FeBIS loading of 8% *w*/*w* (Z_0.2_/CD_0.05_/FeBIS) could be attained by setting different zein and βCD initial concentration in the hydroalcoholic and aqueous phase, respectively, as well as solvent/antisolvent ratio as detailed in 2.4. As shown in Figure 4b, the increase of theoretical loading slightly increased the size of the pseudolatex formed. 

Both FeBIS-containing pseudolatexes were processed by spray-drying, and powders with different yields were collected. For MP_Z_2_/CD_0.5_/FeBIS, the yield remained very high (88%) while product recovered for MP_Z_0.2_/CD_0.05_/FeBIS was lower (45%). SEM images in Figure 4c,d show that micro-sized powders with irregular shape were obtained and, remarkably, a very homogeneous distribution of iron in the matrix was attained.

The Carr’s Index calculated from the bulk/tapped density was 22 ± 0.1% for MP_Z_2_/CD_0.5_/FeBIS and 27 ± 1.8% for MP_Z_0.2_/CD_0.05_/FeBIS (FeBIS-loaded micropowders), whereas it was 37 ± 8.0% for MP_Z_2_/CD_0.5_ and 31 ± 1.7% for MP_Z_0.2_/CD_0.05_ (unloaded micropowders). Significantly higher flowability was found for FeBIS-loaded micropowders (MP_Z_2_/CD_0.5_/FeBIS vs. MP_Z_2_/CD_0.5_ and MP_Z_0.2_/CD_0.05_/FeBIS vs. MP_Z_0.2_/CD_0.05_, *p* < 0.005).

During spray-drying of a multicomponent feeding liquid, the final ratio between components can be altered due to a preferential loss of one component over the others. To check that the amount of zein was maintained throughout the entire drying process, that is to compare theoretical zein employed initially to prepare pseudolatex, and that found in the final micropowders, nitrogen content in the micropowder was assessed through Kjeldahl. The % *N* in zein as raw material was 13.82% in line with values reported in the technical data sheet (13–16%). This value corresponded to a measured % protein of 86.39 calculated on a dry basis (82–100% in the datasheet). As reported in Table 1, values of % *N* and % protein in the micropowder made only with zein were close to those of raw zein, demonstrating that the method can be applied to quantify the protein amount also after spray-drying. For micropowders of zein/βCD or zein/βCD/FeBIS, the theoretical protein content of zein was lower due to the presence of βCD or βCD/FeBIS. The % *N* and % protein in the micropowder made with different amounts of βCD (Table S3) also demonstrated that the actual zein content was always comparable to the theoretical value.

### 3.5. Solid State Interactions in the Micropowders

The micropowders were analysed in the solid state by ATR-FITR, TGA and DSC to investigate the interaction between zein, βCD and FeBIS. Analysis of βCD, FeBIS and zein (raw materials) was also carried out. 

**Figure 5:**
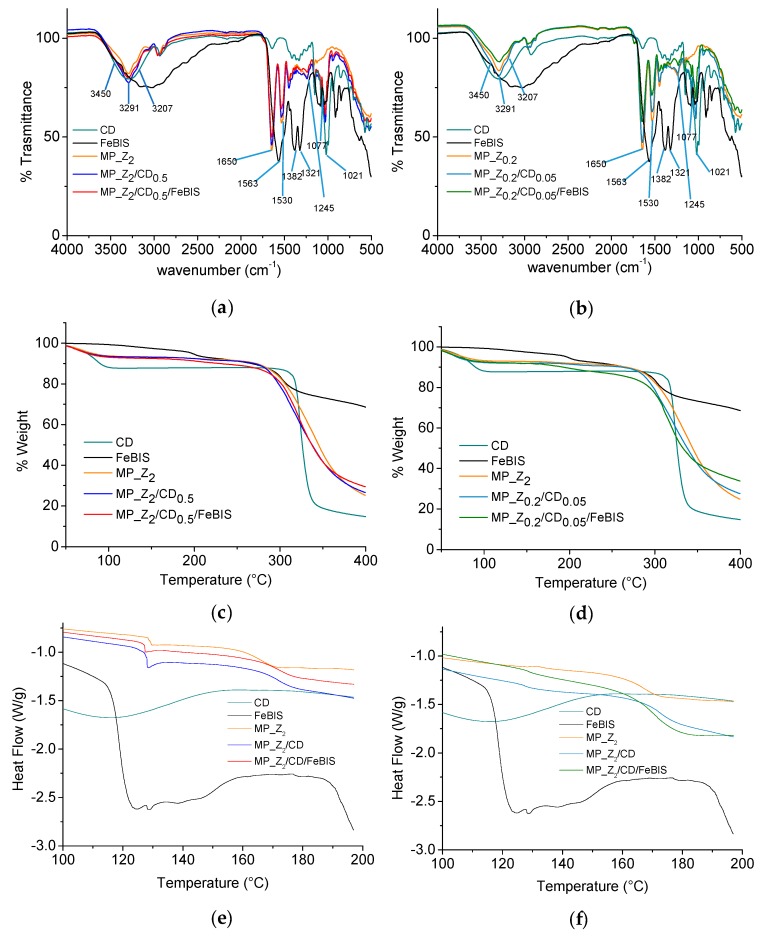
Fourier transform infrared (FTIR) spectra (**a**,**b**), thermogravimetric analysis (TGA) thermograms (**c**,**d**), and differential scanning calorimetry (DSC) profiles (second run) (**e**,**f**) for micropowders prepared at FeBIS theoretical loading of 2% (**a**,**c**,**e**) and 8% *w*/*w* (**b**,**d**,**f**) with corresponding unloaded micropowders, βCD and FeBIS (raw materials).

Attenuated total reflectance–Fourier transform infrared (ATR-FTIR) spectra of micropowders at different FeBIS loading and corresponding unloaded micropowders, βCD and FeBIS (raw materials) were collected over the range of 4000–500 cm^−1^ (Figure 5a,b). Overall, the broad band between 3200 and 3400 cm^−1^ due to hydrogen bonds can be attributed to complex vibrational stretching, associated with free, inter- and intramolecular bound hydroxyl groups, including contributions from primary amides of zein at 3207 and 3450 cm^−1^ [31]. Furthermore, characteristic FTIR bands for proteins with amide I at 1650 cm^−1^, amide II at 1530–50 cm^−1^, and a set of weaker bands that represent amide III vibration modes centred at 1245 cm^−1^ are present (Supplementary information, Figure S2). For the βCD-containing micropowders, the spectrum trend remains similar independently of the presence of FeBIS and amount loaded. This trend is expected since βCD should complex the hydrophobic side chains of zein amino acid sequence. On the other hand, a shift of the bands from 1021 to 1030 and from 1077 to 1080 cm^−1^ (C–O and CO/CC stretches, respectively) are observed 

The thermogravimetric analysis (TGA) of micropowders at different FeBIS loading and corresponding unloaded micropowders, βCD and FeBIS was carried out to monitor the mass loss of the products at an increasing temperature from 40 to 400 °C (Figure 5c,d). TGA for raw zein is reported in supplementary material, Figure S3. The micropowder composition did not significantly affect curve trends. It is worth to notice, however, an incipit of degradation at around 150 °C in the case of MP_Z_0.2_/CD_0.05_/FeBIS, which loads the highest amount of FeBIS. The water content, which corresponds to the initial weight loss until around 100 °C, was found to be the same for all the samples (below 10%).

DSC analysis of the micropowders was run to highlight the occurrence of interactions at a molecular level between the components. Thermograms of the second heating run of micropowders at different FeBIS loading and corresponding unloaded micropowders, βCD and FeBIS are reported in Figure 5e,f whereas glass transition temperature (Tg) values are summarised in Table S4. DSC thermograms in the range 30–200 °C and corresponding Tg values (Supplementary information Figure S4 and Table S5, respectively) are also reported. Tg of zein (Tg1) moves to slightly higher values in the micropowders containing βCD and βCD/FeBIS. The raw βCD shows a broad Tg at 84 °C, according to the literature [32], which is no more visible in zein-based micropowders, confirming strong interactions with the protein. Interestingly, another glass transition at 128 °C (Tg2) was detected in all MP_Z_2_ formulations and, at a lower extent, in MP_Z_0.2_. The transition is accompanied by a relaxation enthalpy well evident in MP_Z_2_/CD_0.5_ and MP_Z_2_/CD_0.5_/FeBIS.

Furthermore, to investigate the melting behaviour of FeBIS in the complex, DSC measurements were acquired up to 250 °C for raw FeBIS and micropowders containing FeBIS (Supplementary information, Figure S5). The thermogram of FeBIS taken in the first heating run shows a broad endotherm centred at around 75 °C, attributable to water evaporation, and two endotherms at 125 °C and 220 °C, which can be reasonably attributed to the melting of some component of raw FeBIS (reasonably, citric acid tert-butyl ester, a mono-ester derivative of citric acid) and the melting of FeBIS, respectively. The broad transitions in the range 150–180 °C are related to degradation as found by TGA. Thermograms of MP_Z_2_/CD_0.5_/FeBIS and MP_Z_0.2_/CD_0.05_/FeBIS show, along with an expected remarkable increase of water evaporation, the absence of FeBIS melting endotherm at 220 °C, pointing out the formation of an amorphous complex. 

### 3.6. Release of FeBIS from Micropowders

**Figure 6:**
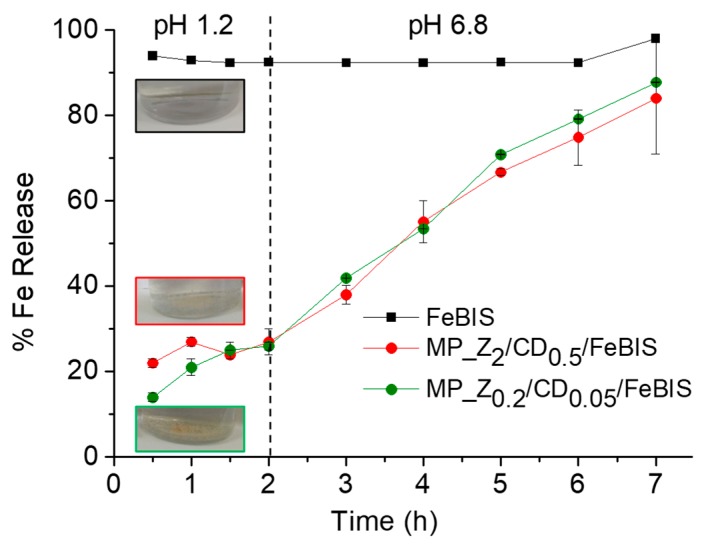
Release of iron from micropowders prepared at FeBIS theoretical loading of 2% (red) and 8% *w*/*w* (green) in simulated gastric fluid (pH 1.2) and simulated intestinal fluid (pH 6.8). Free FeBIS is reported as control. Data are the mean of three separate experiments ± SD.

The release of FeBIS was studied in simulated gastric fluid (SGF) pH 1.2 and simulated intestinal fluid (SIF) pH 6.8, both supplemented with enzymes. As shown in Figure 6, the micropowders loaded with different amount of FeBIS released an amount of total iron (Fe^2+^/Fe^3+^) below 30% after 2 h in SGF while free FeBIS was immediately dissolved, as expected for a water-soluble compound. When the medium was changed to SIF, the iron release occurred at a sustained and constant rate, reaching 80% in 7 h. 

## 4. Discussion

Although food supplements market is expanding very fast, some critical aspects like absorption and bioavailability of bioactive compounds, as well as stability and shelf-life of products have been poorly addressed so far. Thus, there is proliferating research in this field focused on the development of novel advanced strategies to obtain “high performance” products that may adequately fulfil biological requirements and patient’s needs [2]. Given the limited number of materials useful to design a delivery platform for nutrients, the rational combination of raw materials already approved as food ingredients and their processing through nano-micro technology can offer an unprecedented tool for innovation. 

The strategy proposed here consists in processing the hydrophobic plant-based protein zein with the cyclic oligosaccharide βCD and overall aimed to obtain a powder formulation delivering micronutrients. Processing of powders was attempted with a two-step method with the potential of industrial scale-up, where a zein-based pseudolatex incorporating the bioactive component is then processed by spray-drying giving a final solid product with controlled-release features. As a model compound, we selected FeBIS, which is the iron source with the highest oral bioavailability. It is employed in different patient population suffering anaemia (about 25% of the global population) and the fact that there is a rising demand for side-effects free iron supplement products. 

In the first part of the study, we focused on the protein platform and studied the effect of βCD addition to nanoprecipitated zein (pseudolatexes). We highlighted that host–guest interactions between zein and βCD occurred both in solution and in the solid state. Fluorescence analysis of zein and zein/βCD in ethanol/water solutions clearly showed that βCD interacts with the protein, likely altering its conformation. Since zein contains a high amount of tyrosine residues (approx. 5.0% *w/w*) while the level of tryptophan residues is insignificant [28], it is logical to assume that tyrosine is involved in such interaction with βCD. 

On the basis of this preliminary observation, we tried to understand how zein concentration and βCD addition affected pseudolatex size and polydispersity. We found that size and polydispersity of the pseudolatex increased as zein concentration did, while at a fixed zein concentration, βCD addition in the antisolvent increased mean size up to a βCD concentration of 1% *w*/*v*, suggesting that the incorporation of βCD in the zein solid matrix during solvent diffusion process reached a plateau.

The next step of the study consisted in optimising the drying process of the zein/βCD pseudolatex. The modulation of the *t* outlet below Tg of βCD was found crucial to reduce the humidity of the drying gas, thus avoiding the formation of a sticky product and overall increasing the powder yield. NIR analysis on spray-dried powders showed a shift in the spectral bands of powders at increasing βCD amount employed in the pseudolatex preparation, its extent being dependent on the amount of βCD in the formulation. Thus, it could be hypothesised that some chemical groups were involved in the formation of a supramolecular zein/βCD complex. In fact, βCD was partly incorporated inside the obtained particles (around 50%), which further demonstrated that spray-dried powders were constituted by a mixture of zein/βCD and βCD particles.

For FeBIS entrapment in the micropowder, we selected zein and βCD concentrations able (1) to form a pseudolatex withstanding size criteria to be spray-dried and (2) prepared at a high solid content to make the spray-drying process shorter. In parallel, we also tried to maximise FeBIS loading, which is a critical requirement when developing a successful drug delivery strategy. Loading should be as high as possible to reduce the amount of both carrier material employed for production and amount of processed product needed in the final dosage form.

When FeBIS was introduced in the PL_Z_2_/CD_0.5_ formulation, FeBIS theoretical loading could not be higher than 2%. To increase further loading, zein and βCD concentrations needed to be decreased ten times. Although the total solid content was lower, theoretical loading was increased up to 8%. For both pseudolatexes at different theoretical loadings, their size and PI were unchanged as compared with unloaded systems. The yields of FeBIS powders was different, lower in the case of 8% theoretical loading of FeBIS, which is expected if considering that solid content of the corresponding feeding liquid is lower (2.55 and 0.45% *w*/*v* for iron theoretical loading of 2% and 8%, respectively). FeBIS micron-sized powders showed an irregular shape, as often found for spray-dried products. FeBIS-loaded micropowders flowed better as compared to the unloaded counterparts and, remarkably, showed a very homogeneous distribution of iron as evidenced by elemental map analysis through SEM. In fact, the evaluation of N % (i.e., protein %) by Kjeldhal showed that the actual amount of zein in the micropowders was close to the theoretical zein value for all the powders. Thus, the spray-drying process did not alter the original ratios of the components in the formulation, which can be a drawback when processing multicomponent systems. 

The interactions between zein, βCD, and FeBIS were investigated through different solid-state techniques to go in depth in the properties of micropowders. ATR-FTIR spectra of micropowders suggested that interaction between zein and βCD involves the hydrophobic side chains of zein amino acid sequence, while novel zein/βCD and βCD/FeBIS interactions take place [24]. In particular, the characteristic bands related to FeBIS are no more visible in the spectra, and this effect could be attributed to its interaction with βCD. βCD/FeBIS interactions would hinder these molecular vibrations, consequently diminishing the intensities of its absorption bands [24]. 

The micropowder composition did not significantly affect TGA trends, although an incipit of degradation at around 150 °C was found for the micropowder loading the highest amount of FeBIS. This effect can be attributed to the loss of volatile compounds in FeBIS samples, which contains labile components, and is recommended to be processed below 153 °C.

DSC also allowed the study of the occurrence of interactions at a molecular level between components. Tg of zein moves to slightly higher values in the micropowders containing βCD and βCD/FeBIS, likely due to hydrogen bond formation and polar interactions with βCD, which decrease chain mobility. The disappearance of the broad Tg at 84 °C for raw βCD in zein-based micropowders, confirms its strong interactions with the protein. The presence of a Tg2 is evident in all MP_Z_2_ formulations and, at a lower extent, in MP_Z_0.2_ together with a relaxation enthalpy (well visible in MP_Z_2_/CD_0.5_ and MP_Z_2_/CD_0.5_/FeBIS). Since Tg2 is present in spray-dried zein while it is absent in raw zein, it is supposed that the spray-drying process causes a separation between two fractions of zein, resulting in the appearance of the second Tg. This effect could be reasonably due to the presence of zein in different fractions. The relaxation enthalpy indicates that this second phase is in a *nonequilibrium* conformation, where chains have been “frozen” into high-energy conformations because of fast water evaporation during the spray-drying process. The absence of FeBIS melting endotherm at 220 °C in the micropowders suggests its complete amorphization in the matrix. 

Finally, limited FeBIS release in SGF further demonstrated that FeBIS was entrapped in the zein/βCD matrix. The protection of FeBIS can be useful to protect the chelate from dissociation at the low pH values in the stomach. FeBIS is an iron chelate with pH-dependent stability. At acidic pH values, dissociation of the chelate occurs due to the increase of H^+^ concentration, which results in significant ferrous glycinate destruction [33] allowing free iron ions interaction with physiologically relevant anions with high affinity for iron (i.e., phosphates). Furthermore, FeBIS protection may improve iron gastric tolerability and eventual iron precipitation in the gastric mucosa [34], which is of utmost importance if considering the prolonged use of food supplements. In fact, it has been demonstrated that a severe gastric effect occurs in humans consuming iron tablets, with iron deposits found in the stomach causing mucosal erosions [35]. 

Nevertheless, iron was released at a constant rate until 7 h in SIF, demonstrating the sustained-release features of the platform also for a very hydrophilic molecule like FeBIS. Similar results were obtained with spray-dried powders obtained with a procedure similar to that proposed here [36]. The mechanism of release was closely related to water uptake of micropowders which activates FeBIS solubilisation in the matrix. A combination of Fickian diffusion and erosion of the matrix was hypothesised [36]. Since zein is equally ionised at both pH values tested here [37], no relevant difference in water uptake and, in turn, on release rate is found. The impact of an extended and pH-independent FeBIS release on cell internalization and bioavailability cannot be derived a priori and will be the next step in our research activity. 

## 5. Conclusions

In this work, we propose zein/beta-cyclodextrin powders as a delivery platform for bioactive compounds with possible application in food supplements. A two-step nano-in-micro strategy is set up where nanometer-size zein is formed in a first step and then spray-dried to form a micron-sized powder. We demonstrate the crucial role of beta-cyclodextrin as a helping excipient to process zein by spray-drying and to entrap a hydrophilic iron chelate effectively. Studies in the solid state have highlighted that βCD partially interacts with zein and in so doing it increases production yields of the spray-drying process, which is very relevant, taking into account an industrial scale-up. Entrapment of FeBIS in the micropowder is feasible only when βCD is employed in the first preparation step of the nano-in-micro process where modulation of component amount/volume phase ratio can allow achievement of different FeBIS loadings. FeBIS acquires desirable gastro resistance when formulated as micropowders, which avoids chelate destruction occurring at the low pH values in the stomach. In perspective, zein/βCD/FeBIS micropowders could allow efficient absorption of FeBIS in the intestine and can be considered as an innovative strategy for efficient delivery in the body.

## Figures and Tables

**Table 1:** Nitrogen and protein content of zein micropowders.

Batch	% N (±SD) ^a^	% Protein (±SD) ^b^	% Theoretical Protein ^c^
MP_Z_2_	13.67 ± 0.43	85.46 ± 2.69	86
MP_Z_2_/CD_0.50_	11.53 ± 0.05	72.05 ± 0.28	80
MP_Z_2_/CD_0.5_/FeBIS	11.30 ± 0.01	69.91 ± 0.01	78
MP_Z_0.2_	13.89 ± 0.05	86.81 ± 0.29	86
MP_Z_0.2_/CD_0.05_	11.25 ± 0.01	70.34 ± 0.08	80
MP_Z_0.2_/CD_0.05_/FeBIS	11.13 ± 0.02	69.57 ± 0.12	74

^a,b^ calculated as reported in 2.9, ^c^ calculated from the ratio between the mass of zein and the mass of total components used to prepare pseudolatex × 100.

## Supplementary Materials

The following are available online at https://www.mdpi.com/1999-4923/12/1/60/s1, Figure S1: UV and emission spectra of hydroalcoholic solutions containing zein (20 μg/mL) and βCD (0, 2 and 5 μg/mL), Figure S2: FTIR spectra of zein (raw material), Figure S3: TGA thermograms of zein (raw material), Figure S4: Second run thermograms of zein, βCD and FeBIS (raw materials), Figure S5: DSC profiles (first run) of FeBIS-loaded micropowders and raw FeBIS, Table S1: Code and corresponding compositions of zein-based micropowders prepared from different pseudolatex, Table S2: Set-up of spray drying conditions for MP_Z_2_ powders, Table S3: Nitrogen/protein content of zein micropowders using as reference standard zein raw material, Table S4: Glass transition temperatures (Tg) of zein-based micropowders taken in the second heating run, Table S5: Glass transition temperatures (Tg1) of raw materials.
