# Supplementary material for: Zein Beta-Cyclodextrin Micropowders for Iron Bisglycinate Delivery
Source: Pharmaceutics. 2020 Jan 11;12(1):60. doi: 10.3390/pharmaceutics12010060 (PMC7023128; doi:10.3390/pharmaceutics12010060)
Supplement: Supplementary file 1 [file pharmaceutics-12-00060-s001.pdf]

pharmaceutics

Supplementary Materials: Zein Beta-cyclodextrin Micropowders for Iron Bisglycinate Delivery

Diletta Esposito, Giovanni Dal Poggetto, Aurélie Demont, Nicolai Kraut, Agnese Miro, Francesca Ungaro, Paola Laurienzo and Fabiana Quaglia

Table S1. Code and corresponding compositions of zein-based micropowders prepared from different pseudolatex.

Powder codes	Pseudolatex Preparation			Powder composition	
	zein % *w/v* [a]	βCD % *w/v* [b]	Antisolvent/ solvent ratio	zein % *w/w*	βCD % *w/w*
MP_Z_2	4	-	1	2	-
$MP\_ Z_2/CD_{0.25}$	4	0.25	1	2	0.25
$MP\_ Z_2/CD_{0.50}$	4	1	1	2	0.50
$MP\_ Z_2/CD_{0.75}$	4	1.5	1	2	0.75
$MP\_ Z_{0.2}$	2	-	5	0.2	-
$MP\_ Z_{0.2}/CD_{0.05}$	2	0.1	5	0.2	0.05

[a, b] % *w/v* components in the PL produced to obtain the correspondent MP.

Table S2. Set-up of spray drying conditions for MP_Z_2 powders.

Aspirator Flow Rate (%)	Pump Speed (%)	T Inlet (°C)	T Outlet [a] (°C)	Yield (%)
90	10	115	60	50
90	10	150	77	59
90	10	180	121	38
90	20	180	91	23

[a] T outlet is consequent to setting the other parameters.

Table S3. Nitrogen/protein content of zein micropowders using as reference standard zein raw material.

Batch	%N (±SD)	% Protein (±SD)	% Theoretical Protein
$MP\_ Z_2/CD_{0.25}$	12.46±0.02	77.87±0.09	86
$MP\ \_Z_2/CD_{0.75}$	10.29±0.05	64.29±0.38	73

Table S4. Glass transition temperatures (Tg) of zein-based micropowders taken in second heating run.

Batch	Tg_1 (°C)	Tg_2 (°C)
MP_Z_2	165.88	128.47
$MP\_ Z_2/CD_{0.50}$	171.64	128.06
$MP\_ Z_2/CD_{0.5}$/FeBIS	172.28	127.35
$MP\_ Z_{0.2}$	166.77	n.d.
$MP\_ Z_{0.2}/CD_{0.05}$	171.48	128.79
$MP\_Z_{0.2}/CD_{0.05}$/ FeBIS	172.60	127.40

Table S5. Glass transition temperatures (Tg1) of raw materials.

Sample	Tg (°C)
Zein	162
βCD	84

S1. Zein/CD Interactions in Solution

The interaction between βCD and zein was studied in solution by spectroscopic analysis. Zein (20 μg/mL) was dissolved in ethanol:water (8:2 *v*/*v*) solution containing different amounts of βCD (0, 2 and 5 μg/mL) under magnetic stirring. UV–vis spectra were recorded in the wavelength range of 200–800 nm on a UV-1800; Shimadzu. The emission fluorescence spectra of the samples were collected at a fixed excitation wavelength (λ_{ex} 278 nm) in the range of 290–450 nm by a spectrofluorimeter (RF-6000, Shimadzu).

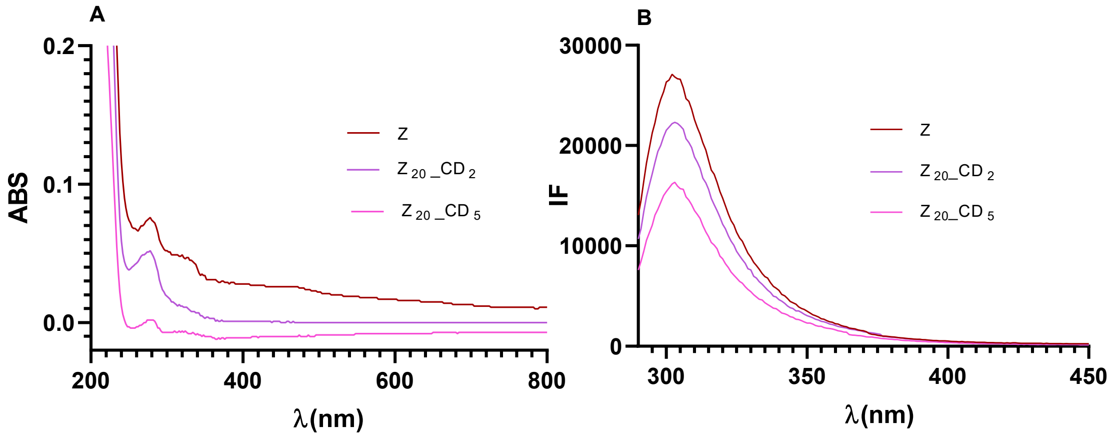

Figure S1. UV and emission spectra of hydroalcoholic solutions containing zein (20 μg/mL) and βCD (0, 2 and 5 μg/mL).

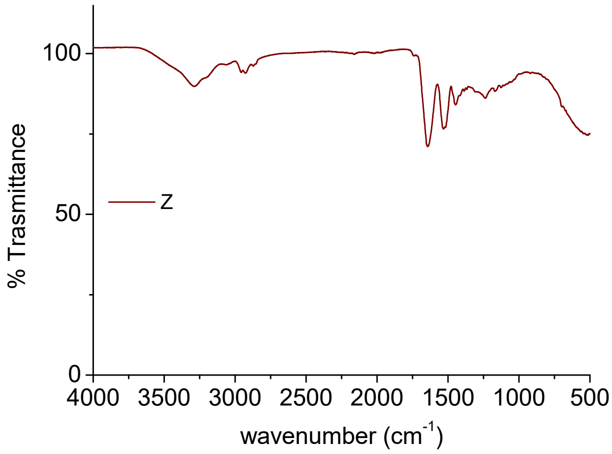

Figure S2. FTIR spectra of zein (raw material).

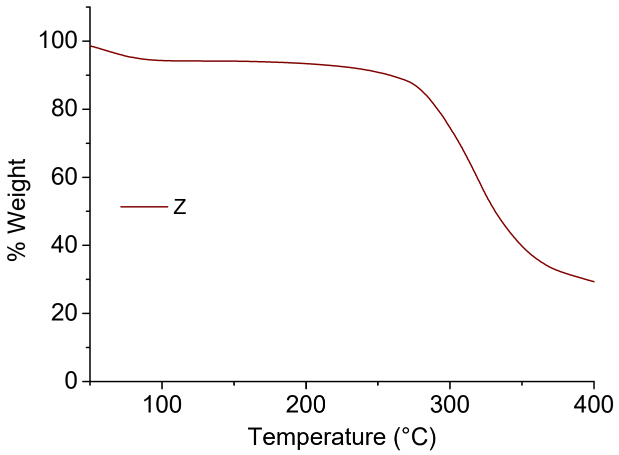

Figure S3. TGA thermograms of zein (raw material).

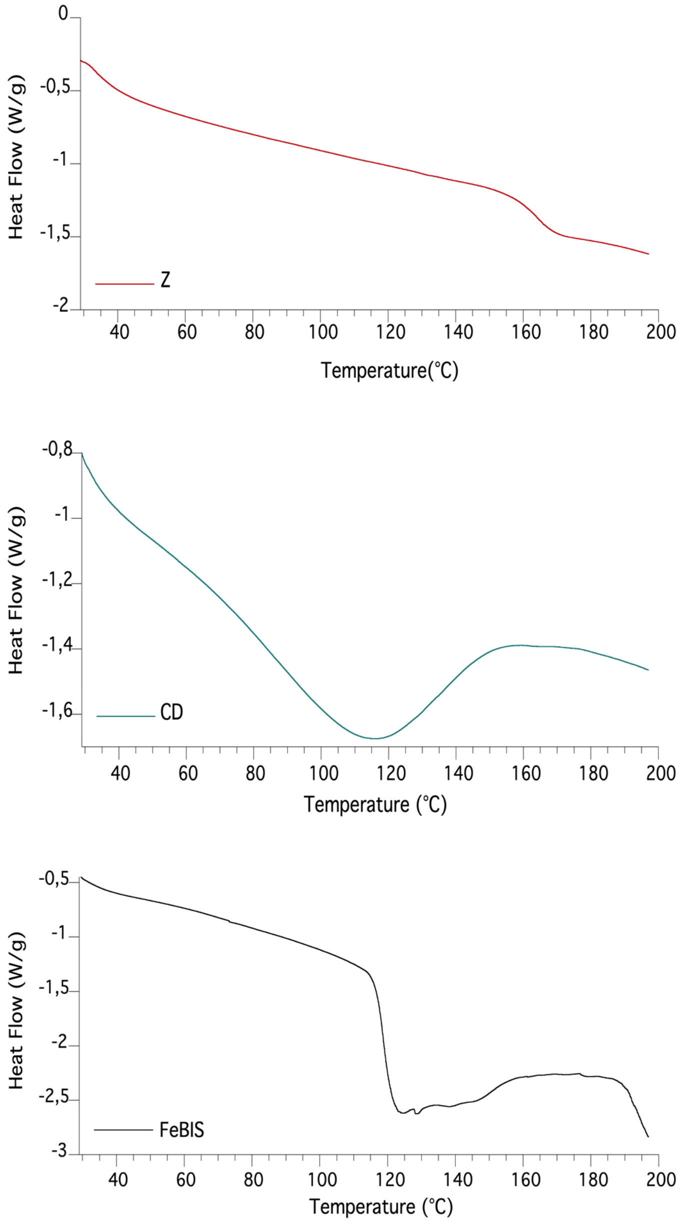

Figure S4. Second run thermograms of zein, βCD and FeBIS (raw materials). The transition observed in the thermogram of FeBIS is related to the incipit of degradation phenomena.

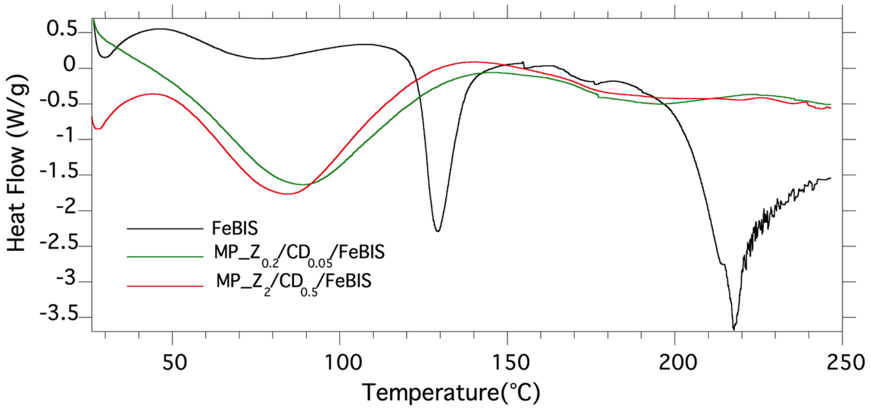

Figure S5. DSC profiles (first run) of FeBIS-loaded micropowders and raw FeBIS.
